# Integrating Machine Learning and Deep Learning for Predicting Non-Surgical Root Canal Treatment Outcomes Using Two-Dimensional Periapical Radiographs

**DOI:** 10.3390/diagnostics15081009

**Published:** 2025-04-16

**Authors:** Catalina Bennasar, Antonio Nadal-Martínez, Sebastiana Arroyo, Yolanda Gonzalez-Cid, Ángel Arturo López-González, Pedro Juan Tárraga

**Affiliations:** 1 Academia Dental de Mallorca (ADEMA), School of Dentistry, University of Balearic Islands, 07122 Palma de Mallorca, Spain; s.arroyo@eua.edu.es; 2 Soft Computing, Image Processing and Aggregation (SCOPIA) Research Group, University of the Balearic Islands (UIB), 07122 Palma de Mallorca, Spain; antonio.nadal@uib.cat; 3 Department of Mathematical Sciences and Informatics, University of the Balearic Islands, 07120 Palma de Mallorca, Spain; yolanda.gonzalez@uib.es; 4 ADEMA-Health Group, University Institute of Health Sciences of Balearic Islands (IUNICS), 02008 Palma de Mallorca, Spain; angarturo@gmail.com; 5 Faculty of Medicine, University of Castilla-La Mancha, 02001 Albacete, Spain; pjtarraga@sescam.jccm.es

**Keywords:** machine learning, deep learning, outcome prediction, non-surgical root canal treatment, apical periodontitis

## Abstract

**Background/Objectives**: In a previous study, we utilized categorical variables and machine learning (ML) algorithms to predict the success of non-surgical root canal treatments (NSRCTs) in apical periodontitis (AP), classifying the outcome as either success (healed) or failure (not healed). Given the importance of radiographic imaging in diagnosis, the present study evaluates the efficacy of deep learning (DL) in predicting NSRCT outcomes using two-dimensional (2D) periapical radiographs, comparing its performance with ML models. **Methods**: The DL model was trained and validated using leave-one-out cross-validation (LOOCV). Its output was incorporated into the set of categorical variables, and the ML study was reproduced using backward stepwise selection (BSS). The chi-square test was applied to assess the association between this new variable and NSRCT outcomes. Finally, after identifying the best-performing method from the ML study reproduction, statistical comparisons were conducted between this method, clinical professionals, and the image-based model using Fisher’s exact test. **Results**: The association study yielded a *p*-value of 0.000000127, highlighting the predictive capability of 2D radiographs. After incorporating the DL-based predictive variable, the ML algorithm that demonstrated the best performance was logistic regression (LR), differing from the previous study, where random forest (RF) was the top performer. When comparing the deep learning–logistic regression (DL-LR) model with the clinician’s prognosis (DP), DL-LR showed superior performance with a statistically significant difference (*p*-value < 0.05) in sensitivity, NPV, and accuracy. The same trend was observed in the DL vs. DP comparison. However, no statistically significant differences were found in the comparisons of RF vs. DL-LR, RF vs. DL, or DL vs. DL-LR. **Conclusions**: The findings of this study suggest that image-based artificial intelligence models exhibit superior predictive capability compared with those relying exclusively on categorical data. Moreover, they outperform clinician prognosis.

## 1. Introduction

Over the past decade, ML and DL have emerged as transformative technologies with significant impacts across multiple scientific disciplines. These techniques, as branches of artificial intelligence (AI), have become essential tools for analyzing large volumes of data, identifying complex patterns, and providing innovative solutions to previously intractable problems. Their relevance has been particularly emphasized in health sciences, including medicine, biology, and, more recently, dentistry, where their application holds the potential to optimize diagnostics, personalize treatments, and improve clinical outcomes.

ML can be defined as a subfield of AI that employs algorithms to enable machines to learn patterns and behaviors from data without being explicitly programmed for each specific task (Samuel, 1959 [1]). Within this field, DL represents a significant evolution, utilizing artificial neural networks with multiple layers capable of learning hierarchical data representations. These deep architectures have proven especially effective in tasks involving large datasets and complex features, such as medical image interpretation, disease prediction, and biological signal analysis [2].

The current utility of ML and DL in health sciences is demonstrated in a wide range of applications, including but not limited to medical image analysis, genomic data mining, drug modeling, and clinical outcome prediction. For instance, in radiology, convolutional neural networks (CNNs) have been successfully employed to detect tumor lesions in computed tomography (CT) and magnetic resonance imaging (MRI) scans, achieving accuracy levels comparable to those of human experts [3]. In genomics, DL algorithms have facilitated the decoding of complex gene interactions, accelerating the development of personalized therapies [4].

In the field of dentistry, the impact of ML and DL is beginning to solidify with promising applications. Dentistry, as a health science, has undergone significant digital transformation in recent years, driven by technologies such as cone-beam computed tomography (CBCT), three-dimensional (3D) printing, and CAD/CAM systems. However, the integration of ML and DL in this domain has opened new avenues for diagnosis, treatment design, and disease monitoring. For example, DL algorithms have been effective in identifying dental caries [5], fractures, periodontal diseases [6], and periapical conditions from digital radiographs, enhancing diagnostic accuracy and reducing variability among professionals [7].

Beyond diagnostics, these technologies are beginning to influence treatment planning and execution. In orthodontics, for example, ML is used to predict tooth movement and optimize the placement of brackets or aligners, leading to more effective and personalized treatments [8]. In implantology, ML models assist in predicting dental implant stability over time by considering factors such as bone density, implant location, and patient characteristics [9]. In endodontics, AI supports professionals by detecting periapical lesions, identifying root fractures, analyzing root canal morphology, predicting retreatment needs, and aiding in regenerative pulpal therapy, all of which contribute to improved diagnostics, treatment planning, and patient care [10,11,12,13,14,15,16].

The future of ML and DL in health sciences, particularly in dentistry, promises to be even more revolutionary. It is anticipated that the combination of these technologies with advanced sensor systems and data from wearable devices will enable continuous, real-time monitoring of oral health. For example, the integration of DL with intraoral connected devices could facilitate the early detection of diseases such as oral cancer through the analysis of salivary biomarkers or intraoral images [17]. Additionally, the development of explainable AI (XAI) systems could address one of the most pressing current challenges: the need to provide clear and interpretable explanations of algorithm-generated predictions, fostering clinical acceptance and ethical use [18].

Despite these promises, the implementation of ML and DL in clinical practice faces significant challenges that must be addressed to ensure success. These include the need for large volumes of high-quality data for model training, ethical and legal concerns related to data privacy, and the necessity of educating healthcare professionals in the use of these technologies. These challenges highlight the importance of interdisciplinary collaboration involving researchers, technology developers, clinicians, and policymakers [19,20].

## 2. Study Objectives

To evaluate DL as an additional variable in an ML study for predicting NSRCTs in cases of AP. This study aims to determine the extent to which deep neural networks can predict the outcome of NSRCTs in teeth with apical periodontitis using digital periapical radiographs of confirmed AP diagnoses.

## 3. Materials and Methods

### 3.1. Sample Selection

A retrospective study was conducted based on the analysis of clinical records of patients with AP who underwent NSRCTs for the first time (not retreatments). Cases were randomly selected from the database of a private clinic in Mallorca, Spain. Only patients without reported systemic diseases [21] who received treatment for the first time and whose records included the following were included:

A comprehensive medical and dental history with general, facial, and oral inspection reports, as well as dental percussion and palpation examinations.

Results of complementary tests, such as thermal sensitivity testing using an ice pencil and periapical radiography.

A follow-up period of at least nine years, starting six months after treatment, with documented evaluations of lesion recovery, categorizing cases as successful (0: there are no symptoms or indications for further treatments, and the lesion disappears after NSRCT) or failed (1: the failure occurs when either the clinical or radiographic outcome fails).

Radiographs were acquired using an X Mind Unity Acteon Satelec system with a focal point of 0.4 mm, at 70 kV and 7 mA, employing a Carestream 6100 digital system with a resolution of 15 LP/mm. The bisecting angle technique was used with a Rinn XCD (Dentsply) positioner (Dentsply, Charlotte, NC, USA) [22]. Patients with vertical root fractures or teeth without sufficient ferrule structure for subsequent restoration were excluded.

Due to this filtering process, the final number of patients included in this study was reduced to 119. Patient consent was waived due to the inability to identify participants in the database. The Research Ethics Committee of the Balearic Islands (IB4015/19IP) approved this study.

### 3.2. Intervention Procedure

The 119 patients with confirmed AP, for whom eight preoperative domain variables were observed as per a recommended data collection template (DCT) for endodontic treatment evaluation studies [23,24,25], underwent standardized endodontic treatment performed by the same endodontist using identical materials and procedures. The following phases were followed:

Local anesthesia administration and rubber dam placement.

Chamber access and pre-enlargement of the coronal third, followed by apical third negotiation.

Working length determination using a Morita apex locator (Morita, Tokyo, Japan) and radiographic confirmation. The working length was always set at the radiographic apex level.

Instrumentation with K3 (SybronEndo, Orange, CA, USA) and Protaper Gold (Dentsply Maillefer, Woodbridge, ON, USA) rotary systems, complemented with manual instruments.

Irrigation with EDTA and 5.25% sodium hypochlorite.

Obturation using the warm vertical condensation technique with AH Plus sealer.

Following treatment completion, cases were radiographically evaluated to rule out overfills or obturation defects.

### 3.3. Machine Learning and Deep Learning Analysis

To compare DL with ML models, a previous study, “Second Opinion for NSRCT Prognosis Using Machine Learning Models” [26], was utilized, in which logistic regression (LR), random forest (RF), naive Bayes (NB), and k-nearest neighbor (KNN) algorithms were applied. The RF algorithm demonstrated the best performance.

**Figure 1:**
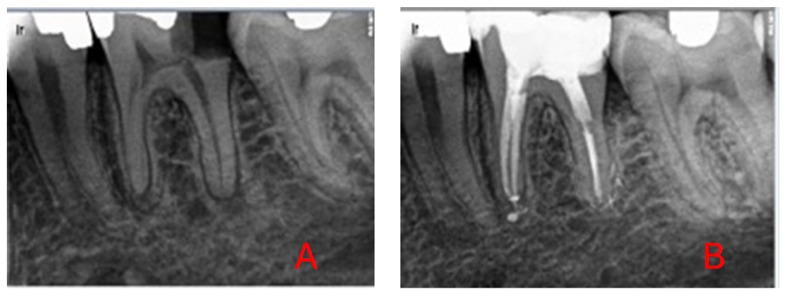
(**A**) AP in tooth 36; (**B**) three-year follow-up: healed.

**Figure 2:**
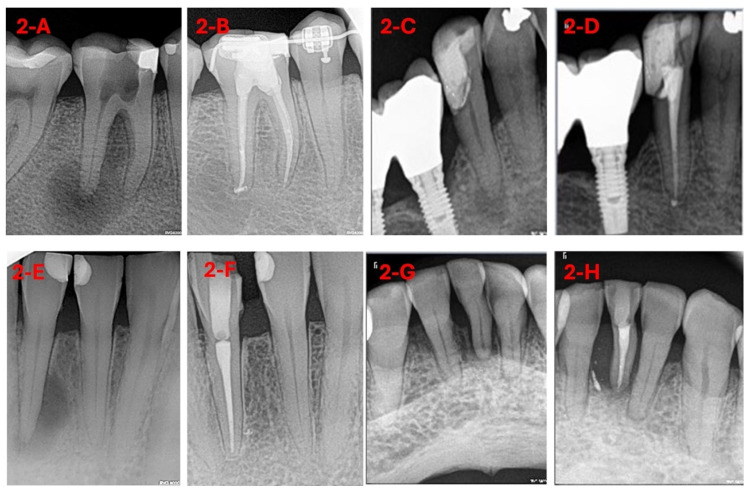
(**A**) AP in tooth 46; (**B**) one-year follow-up: healed; (**C**) AP in tooth 45; (**D**) four-year follow-up: healed; (**E**) AP in tooth 41; (**F**) three-year follow-up: healed; (**G**) AP in tooth 31; (**H**) two-year follow-up: not healed.

All periapical radiographs, 108 in total since due to geometric distortion or anatomical noise 11 were excluded, used for DL model training were preoperative images obtained prior to NSRCT, labeled based on post-treatment follow-up outcomes (healed: 0/not healed: 1) after a minimum of 9 years (Figure 1 and Figure 2).

**Figure 3:**
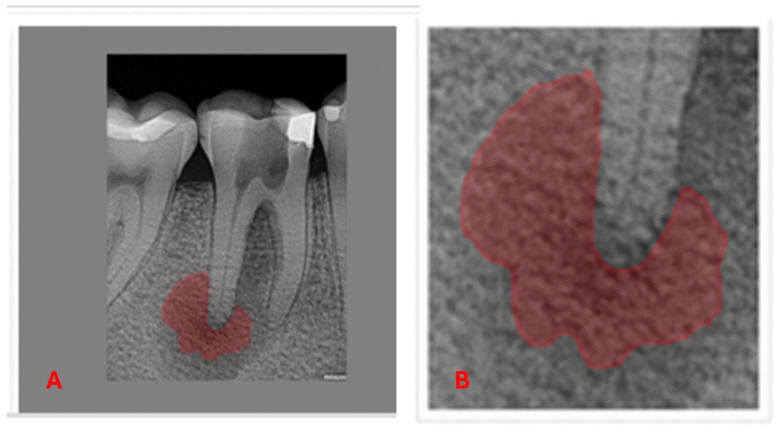
Demarcation of apical periodontitis. (**A**) demarcation of the AP in the vision of the tooth as a whole. (**B**) demarcation of the AP in the apical area of the distal root of the same tooth.

The AnotIA software was utilized for the precise segmentation of diagnostic 2D periapical radiographic images of AP, assigning labels to facilitate subsequent analysis (Figure 3). Although the segmented regions produced by AnotIA were not used as direct input for the DL model (i.e., no semantic segmentation masks were provided to the network), these annotations were employed during model development and testing to ensure that the network’s focus aligned with clinically relevant areas. Specifically, the marked regions of apical lesions were used to visually confirm that the model’s predictions and activation maps corresponded to diagnostically meaningful locations. This validation strategy contributed to increasing the model’s interpretability and may serve as a preliminary step toward incorporating explainable AI (XAI) techniques in future research.

**Figure 4:**
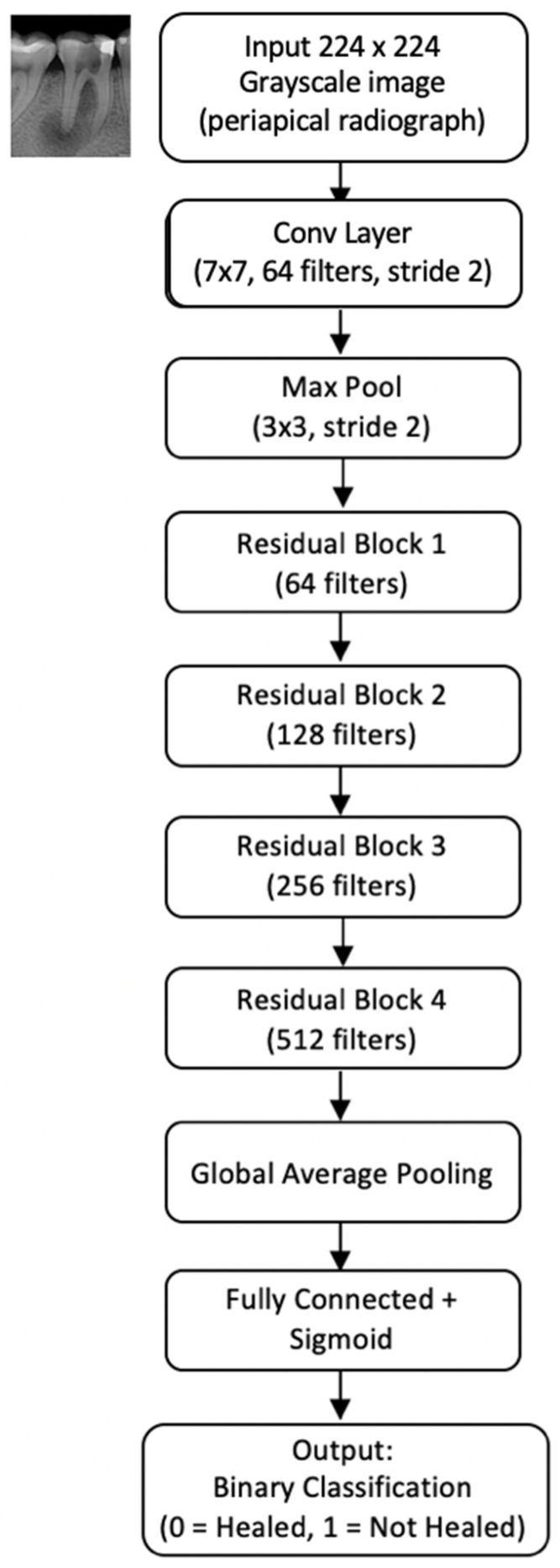
ResNet-18 architecture employed for binary classification of non-surgical root canal treatment (NSRCT) outcomes. The model receives a 224 × 224 grayscale periapical radiograph as input. It processes the image through a series of convolutional layers and residual blocks with increasing filter sizes (64, 128, 256, and 512). A Global Average Pooling layer precedes the Fully Connected Layer, which outputs a binary classification (0: Healed, 1: Not Healed). This architecture enables the model to capture hierarchical features relevant to apical periodontitis prognosis.

Diagnostic two-dimensional AP images were employed to train a convolutional neural network based on the ResNet-18 architecture, a deep 18-layer network designed for recognizing complex patterns in medical images (Figure 4). This architecture has demonstrated efficacy in various AI applications in dentistry [27,28,29,30,31,32,33] due to its residual connections, which facilitate deep network training and mitigate accuracy degradation issues.

The training and validation process of DL follows the same LOOCV scheme used in the evaluation of ML algorithms, including logistic regression (LR), random forest (RF), naive Bayes (NB), and K nearest neighbors (KNN) [26,34]. In this approach, the DL treatment prognosis for each patient is determined by training the model with the images of the remaining patients.

The use of LOOCV is particularly valuable for assessing the performance of artificial intelligence models, as it systematically excludes one data point from the training set, using it as a validation or test instance. Subsequently, a predictive value is generated for the excluded data, and this process is repeated as many times as elements are in the training set. Finally, the predicted values for each excluded data point are compared with the observed values, allowing for a rigorous evaluation of model performance.

In the ML study, once the variable “Prediction by DL” was incorporated into each of the models, the LOOCV scheme was applied again. For variable selection, the Backward Stepwise Selection (BSS) technique was used [34], a commonly employed method for identifying the most relevant features in predictive models.

This study aims to provide evidence regarding the predictive capability of DL in NSRCT prognosis, comparing its performance with conventional ML models and validating its applicability in clinical settings.

### 3.4. Statistical Analysis

For statistical analysis, we relied on the results obtained in our previous study, where a set of preoperative patient variables, both clinical and demographic, were used as explanatory covariates in various ML models to predict treatment outcomes [26]. In the present study, we included an additional explanatory covariate: the treatment outcome prediction obtained by applying convolutional networks to the diagnostic images of 108 patients, training the networks to forecast the prognosis.

After establishing the performance of DL and the best performing ML model a series of statistical comparisons will be conducted.

For this analysis, Fisher’s exact test will be employed, setting a significance level of 0.05. Any result with a *p*-value below 0.05 will be considered statistically significant. The comparisons to be evaluated are as follows:

- Comparison between the best ML model from the previous study [26], random forest (RF), and the combined DL and best performing ML model.
- Comparison between random forest and DL in general: (RF vs. DL).
- Comparison between the clinical professional’s prediction (DP) and the combined DL and best performing ML model.
- Comparison between the clinical professional’s prediction (DP) and the DL model by itself: (DP vs. DL).
- Comparison between the combined DL and best performing ML model and DL model.

These comparisons will assess the relative efficacy of different predictive approaches, providing valuable insights into the applicability of AI models in predicting the success of NSRCT.

## 4. Results

Using the results obtained from the DL study, we applied the chi-square test to detect the association between DL results (DL prediction) and the dentist’s outcome, obtaining a *p*-value of 0.000000127 and an effect size of 0.53 (Table 1) supporting the inclusion of this variable as part of the analysis.

After replicating the study in [26] with this new variable, the best performance was obtained with an LR model, in which the most influential variables were “DL” (predictions generated by DL networks), “Age”, “Smoking”, “Level_Education”, “Periapical” (periapical condition), and “Prognosis”.

The performance of all methods used in this study is presented in Table 2.

Having established the performance of DL and LR, the above-mentioned comparisons were performed.

### 4.1. Comparison Between Random Forest (RF) and the Deep Learning–Logistic Regression Model (DL-LR)

Overall, DL-LR outperformed the best-performing machine learning model from the previous study [26], random forest, achieving sensitivity, specificity, positive predictive value (PPV), negative predictive value (NPV), and accuracy of 0.87, 0.65, 0.79, 0.77, and 0.78, respectively. In comparison, random forest yielded values of 0.83, 0.70, 0.79, 0.74, and 0.77 for the same metrics. However, the differences were not statistically significant, suggesting similar performance between the two models.

### 4.2. Comparison Between Random Forest and Deep Learning in General (RF vs. DL)

The comparative analysis between the overall DL model and random forest showed no statistically significant differences in their performance.

### 4.3. Comparison Between the Clinical Professional’s Prediction (DP) and Deep Learning–Logistic Regression (DP vs. DL-LR)

In the comparison between DL-LR and DP, DL-LR demonstrated better performance in sensitivity, NPV, and accuracy. Using the true positive (TP) and false negative (FN) values from Table 2, a statistically significant difference was observed in the sensitivity of the logistic regression model with DL compared to the professional’s prediction (*p*-value = 0.00041). However, using the false positive (FP) and true negative (TN) values from the same table, no significant differences were found in specificity or PPV. Nevertheless, significant differences were identified in NPV (*p*-value = 0.01563) and accuracy (*p*-value = 0.00253).

### 4.4. Comparison Between the Clinical Professional’s Prediction (DP) and Deep Learning (DL) (DP vs. DL)

Similarly, when comparing the standalone DL model with DP, statistically significant differences were found in sensitivity (*p*-value = 0.00005), NPV (*p*-value = 0.0108), and accuracy (*p*-value = 0.00421), indicating superior performance of DL in these key metrics.

### 4.5. Comparison Between Deep Learning and the Combined Logistic Regression–Deep Learning Model (DL vs. DL-LR)

Finally, when comparing the individual DL model with the logistic regression model supplemented with categorical variables and the output of the DL model (DL-LR), no statistically significant differences were found in any of the evaluated metrics, suggesting equivalent performance between both models.

### 4.6. Interpretation of Statistical Comparisons

Based on statistical comparisons: DP vs. DL, RF vs. DL, and DL-LR vs. DL, the following conclusions can be drawn:

Categorical variables have a lower predictive value compared to image-based models.

Sensitivity, NPV, and accuracy metrics show minimal or non-significant differences between RF vs. DL and DL-LR vs. DL models.

High *p*-values (>0.05) in model comparisons indicate no real difference between categorical data-based approaches.

No clear improvements were found in any metric for RF vs. DL and DL-LR vs. DL comparisons.

In contrast, the DP vs. DL comparison showed significant differences:

DL vs. DP exhibited very low *p*-values (0.00005 in sensitivity, 0.00421 in accuracy, 0.0108 in NPV), suggesting that image-based models (likely represented by DL) have superior predictive power compared with dental professionals (DPs).

Similarly, DP vs. AI-based methods showed significant differences:

DL-LR vs. DP displayed very low *p*-values (0.00041 in sensitivity, 0.00253 in accuracy, 0.01563 in NPV), indicating that AI-based methods (DL-LR) have better predictive value than dental professionals (DPs).

## 5. Discussion

The results obtained in this study, supported by the statistical data collected, highlight the need to compare our AI-based NSRCT prediction for AP with the existing literature to validate our findings scientifically. However, this comparison is challenging due to the limited number of studies dedicated to predicting NSRCT outcomes for apical periodontitis using AI applied to 2D periapical radiographs in endodontics.

AI systems have demonstrated significant advancements in medical imaging, substantially contributing to diagnosis and treatment planning across various specialties. In medicine, convolutional neural networks (CNNs) have been employed for the automatic analysis of pathologies such as breast cancer [35], lung cancer [36,37], and Alzheimer’s disease [38]. In dentistry, AI applications have included dental caries detection [39,40], implant classification [40,41], periodontal bone loss quantification [40,42], and cyst evaluation using various types of radiographs, including periapical, panoramic, cephalometric, and CBCT images [43]. In endodontics, AI has been applied to detect apical periodontitis [7] and C-shaped root canals [44].

Although DL applications in medicine are well established [45], studies on disease and treatment outcome prediction in endodontics remain considerably limited [27,35,36]. In this context, Lee et al. (2023) [27] conducted a study predicting endodontic treatment and retreatment outcomes over a three-year period using 598 preoperative periapical radiographs of single-rooted premolars. Utilizing a ResNet-18 CNN model, trained, validated, and tested, their study focused on two main objectives: detecting various clinical features and predicting treatment outcomes. Their findings confirmed the feasibility of DCNN algorithms for feature detection and endodontic prognosis prediction.

Our study shares the objective of evaluating the predictive capability of endodontic treatments using DL with a ResNet-18 architecture, however, our methodology considers all tooth types, not just single-rooted premolars. The selection of single-rooted premolars in Lee et al.’s study [27] was based on the lower anatomical variability of these teeth compared to incisors or molars, which can present heterogeneous periapical conditions [46,47]. Additionally, all cases analyzed in our study exhibited AP, reducing treatment outcome variability. Unlike Lee et al.’s study [27], our research did not include retreatments, which can influence treatment success rates. Furthermore, our study’s evaluation period was extended to nine years, whereas Lee et al. [27] conducted a three-year follow-up. This distinction is relevant, as short-term evaluations may not fully capture the healing process [47].

A key methodological aspect in endodontic treatment evaluation is the use of the periapical index (PAI) score [48]. In Lee et al.’s study [27], only PAI scores 1, 4, and 5 were considered, omitting stage PAI 3, which reflects bone structural changes with minimal demineralization characteristic of apical periodontitis [49]. In our study, we opted to dichotomize the PAI evaluation to avoid ambiguities. Moreover, our study accounted for working length and obturation type, which are critical parameters influencing treatment success rates [50,51,52].

In a broader context, the literature has explored various AI applications in endodontics. A study employing the AGMB-Transformer model used a dataset of 245 radiographic images of root canal treatments to evaluate its performance in anatomical structure segmentation and outcome classification [53]. Although this study did not focus on treatment prediction, it demonstrated that combining segmentation and classification data significantly improves automated evaluations.

Systematic reviews by Aminoshariae et al. [11], Khanagar et al. [13,16] and Herbst et al. [54] have consolidated knowledge of AI in endodontics, addressing areas such as diagnosis, clinical decision-making, and therapeutic success prediction. However, predicting endodontic treatment outcomes remains an unexplored research gap. Parvathi et al. [10] analyzed AI applications in endodontics, including apical foramen localization, root fracture detection, and retreatment prediction. Campo et al. [55] introduced a case-based reasoning (CBR) system to minimize failed retreatments, however, research addressing NSRCT outcome prediction remains scarce [56].

The use of ResNet-18 architectures in dentistry has proven to be an effective methodology for various applications, including dental caries classification [29], apical periodontitis detection [28], and periodontal disease evaluation [32]. Other studies have employed DL for anatomical structure segmentation [57], predicting inferior alveolar nerve paresthesia after third molar extraction [31], and detecting external root resorptions [30].

Despite advancements in AI applications in endodontics, the current literature presents a shortage of studies focusing on predicting the outcomes of primary endodontic treatments for apical periodontitis. As evidenced by Lee et al. [27] and Yunxiang Li et al. [53], additional studies are imperative. Compared to medicine, where AI has demonstrated significant advancements, efforts in endodontics remain focused on detecting periapical lesions [15,19,28,56,58,59,60], root morphology analysis [15,19,56,58], and retreatment prediction [55,61], leaving considerable room for future research on NSRCT outcome prediction.

## 6. Conclusions

The findings of this study suggest that image-based artificial intelligence models (DL) exhibit superior predictive capability compared with those relying solely on categorical data. Significant improvements in DL were observed compared with professional prognosis (DP), whereas, differences among models utilizing categorical data were minimal or statistically insignificant. This finding supports the hypothesis that the information contained in images provides greater richness and discriminatory power in predicting endodontic treatment success compared with categorical data.

These results reinforce the importance of radiographic analysis in evaluating AP and its potential progression, highlighting the critical role of AI models in optimizing clinical diagnoses and therapeutic decision-making. Additionally, further exploration of hybrid models that integrate categorical and imaging data is recommended to enhance predictive accuracy in endodontics.

## 7. Limitations

Despite the promising findings, this study presents certain limitations that must be considered when interpreting the results. First, the model was developed and validated using a restricted dataset collected from a single institution and obtained using a single radiographic device. This lack of heterogeneity in the sample may affect the generalizability of the results to other populations and clinical settings. Additionally, the limited number of samples available for training could contribute to model overfitting, where the algorithm performs well on the training data but fails to generalize to unseen cases. Although LOOCV was employed to mitigate this risk and maximize data usage, this approach can still yield high variance in performance metrics when applied to small datasets. As a result, the robustness and reliability of the model in broader clinical applications may be limited, underscoring the need for further validation with larger, more diverse cohorts.

Furthermore, the scarcity of previous studies addressing the prediction of the success of NSRCTs for apical periodontitis using artificial intelligence poses a challenge for comparing and validating our findings against the existing literature. The limited availability of specific bibliographic material hinders the direct comparison of our results with other predictive models in endodontics, highlighting the need for further research in this area.

Therefore, we recommend conducting multicenter studies with larger sample sizes and diverse radiographic equipment, as well as integrating complementary clinical data to enhance the applicability of these models in dental practice.

## Figures and Tables

**Table 1:** Variables associated with the results of the previous ML study, incorporating the DL prediction.

Variable	Levels	*p*-Value	Effect Size
Age	15–24; 25–34; 35–44; 45–54; 55–64; ≥65	0.0056	0.372
Highest level of education	Primary; Secondary; Post secondary	0.0016	0.33
Arch	Mandible; Maxilla	0.02	0.21
Smoking	No; Every day; Some days; Former	0.046	0.26
Patient co-operation	No; Yes	0.028	0.21
Pain relieved by	None; Cold; Medication	0.003	0.31
Duration of the pain	Sec; Min; Continuous	0.027	0.245
Periapical	Asymptomatic AP; Symptomatic AP; Chronic Apical Abscess; Acute Apical Abscess	0.01	0.31
Estimated prognosis by clinician	Hopeless; Questionable; Fair; Good; Excellent	0.034	0.29
Prediction by DL	Success; Failure	0.000000127	0.53

**Table 2:** Performance of AI algorithms and the dentist prognosis (DP).

Metric	DP	RF	Logistic Regression (DL-LR)	DL
TP	42	57	57	59
FN	27	12	8	6
FP	21	15	15	18
TN	29	35	28	25
Sensitivity	0.61 (0.48, 0.72)	0.83 (0.72, 0.91)	0.87 (0.77, 0.94)	0.90 (0.80, 0.90)
Specificity	0.58 (0.43, 0.72)	0.7 (0.55, 0.82)	0.65 (0.49, 0.78)	0.58 (0.42, 0.72)
PPV	0.67 (0.54, 0.78)	0.79 (0.68, 0.88)	0.79 (0.67,0.87)	0.76 (0.65, 0.85)
NPV	0.52 (0.38, 0.65)	0.74 (0.6, 0.86)	0.77 (0.60, 0.89)	0.80 (0.62, 0.92)
Accuracy	0.6 (0.5, 0.69)	0.77 (0.69, 0.84)	0.78 (0.69, 0.86)	0.77 (0.68, 0.85)

## Data Availability

Full datasets and R scripts are available upon reasonable request to the corresponding author.
